# Can We Reliably Identify the Pathological Outcomes of Neoadjuvant Chemotherapy in Patients with Breast Cancer? Development and Validation of a Logistic Regression Nomogram Based on Preoperative Factors

**DOI:** 10.1245/s10434-020-09214-x

**Published:** 2020-10-23

**Authors:** Jian Zhang, Linhai Xiao, Shengyu Pu, Yang Liu, Jianjun He, Ke Wang

**Affiliations:** 1 Department of Breast Surgery, The First Affiliated Hospital of Xi’an Jiaotong University, No. 277 Yanta West Road, Xi’an, 710061 China; 2 School of Public Health, Fudan University, No. 130 Dong’an Road, Shanghai, 200032 China

## Abstract

**Background:**

Pathological responses of neoadjuvant chemotherapy (NCT) are associated with survival outcomes in patients with breast cancer. Previous studies constructed models using out-of-date variables to predict pathological outcomes, and lacked external validation, making them unsuitable to guide current clinical practice.

**Objective:**

The aim of this study was to develop and validate a nomogram to predict the objective remission rate (ORR) of NCT based on pretreatment clinicopathological variables.

**Methods:**

Data from 110 patients with breast cancer who received NCT were used to establish and calibrate a nomogram for pathological outcomes based on multivariate logistic regression. The predictive performance of this model was further validated using a second cohort of 55 patients with breast cancer. Discrimination of the prediction model was assessed using an area under the receiver operating characteristic curve (AUC), and calibration was assessed using calibration plots. The diagnostic odds ratio (DOR) was calculated to further evaluate the performance of the nomogram and determine the optimal cut-off value.

**Results:**

The final multivariate regression model included age, NCT cycles, estrogen receptor, human epidermal growth factor receptor 2 (HER2), and lymphovascular invasion. A nomogram was developed as a graphical representation of the model and showed good calibration and discrimination in both sets (an AUC of 0.864 and 0.750 for the training and validation cohorts, respectively). Finally, according to the Youden index and DORs, we assigned an optimal ORR cut-off value of 0.646.

**Conclusion:**

We developed a nomogram to predict the ORR of NCT in patients with breast cancer. Using the nomogram, for patients who are operable and whose ORR is < 0.646, we believe that the benefits of NCT are limited and these patients can be treated directly using surgery.

Breast cancer is the most common malignancy and the second leading cause of cancer death among women in America. The incidence of breast cancer is increasing.^1^ Neoadjuvant chemotherapy (NCT), which is increasingly offered to patients with breast cancer, may be used to reduce the tumor burden to enable breast-conserving surgery (BCS), and provides an opportunity to assess the response to treatment using an in vivo chemosensitivity test.^2^^,^^3^ Patients achieving a pathological complete response (pCR) following NCT are associated with significantly better event-free survival (EFS) and overall survival (OS), especially for patients with human epidermal growth factor receptor 2 (HER2)-positive (HER2+) and triple-negative breast cancer.^4^^,^^5^ However, only 30–73% of patients achieve pCR,^6^^,^^7^ considering that the application of immunotherapy in NCT is still in the research stage, meaning that the majority of patients do not achieve pCR. Therefore, the development of a practical tool to predict the pathological response in patients with breast cancer after NCT is necessary.

Previous studies suggested that certain baseline clinicopathological features could predict the efficacy of NCT for breast cancer, such as hormone receptor (HR) status, histological grade, proliferation index, tumor size, and laboratory indicators. However, the results of different studies varied, indicating the inability of a single factor to predict the efficacy of chemotherapy for breast cancer. In addition, previous studies constructed models using out-of-date variables to predict pathological responses (such as the cut-off value that determines HR positivity and the application of targeted therapy in NCT), and lacked external validation, making them unsuitable to guide current clinical practice^8^^–^12.

Nomograms that integrate clinical and pathological variables using multiple logistic regression have been shown to enable more accurate prediction for individual patients in diverse types of tumors.^13^^–^16 However, there are few well-designed nomograms to predict the probability of pathological outcomes in the literature, and the implications of providing a detailed probability of pathological response in patients who receive NCT are not well-established. Therefore, in the present study, we established a nomogram based on pretreatment clinicopathological variables to calculate the likelihood that patients with breast cancer would benefit from NCT.

## Materials and Methods

### Patient Population

**Fig. 1:**
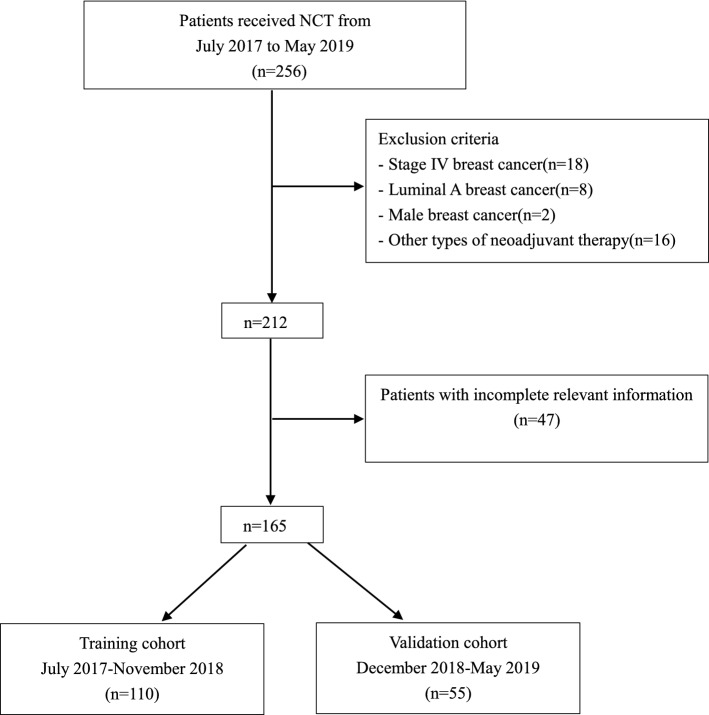
Study design. A total of 165 patients with breast cancer with complete relevant information and who received NCT were enrolled in this study. *NCT* neoadjuvant chemotherapy

This study included 256 patients with operable breast cancer who received NCT between July 2017 and May 2019 at the First Affiliated Hospital of Xi’an Jiaotong University. All patients were diagnosed with invasive breast cancer using hollow needle biopsy before chemotherapy, and received two to six cycles of NCT before surgery. The chemotherapy regimens were based on anthracyclines and/or taxanes, including anthracycline plus cyclophosphamide, followed by anthracycline-based, taxane-based, or trastuzumab regimens. Treatment was suspended when intolerable toxicity, disease progression, or other conditions that were considered unsuitable to continue chemotherapy occurred. Modified radical mastectomy, breast conservation, or breast reconstruction, combined with sentinel lymph node biopsy or axillary lymph node dissection, were performed within 1 month after the completion of NCT. Based on the clinical evaluation and postoperative pathology report before NCT, we determined if further chemotherapy, radiotherapy, and endocrine therapy were needed after surgery. For HER2+ patients, anti-HER2-targeted therapy was required for 1 year. The exclusion criteria included (1) stage IV breast cancer with distant metastasis; (2) luminal A breast cancer; (3) male breast cancer; and (4) acceptance of other types of new adjuvant therapy, including endocrine therapy and radiotherapy. Finally, 165 patients with breast cancer with complete relevant information who received NCT were enrolled. The eligible patients were divided into a training cohort (nomogram construction) and a validation cohort (nomogram validation). The training cohort consisted of 110 patients with breast cancer who received NCT between July 2017 and November 2018, while the validation cohort consisted of 55 patients with breast cancer who received NCT between December 2018 and May 2019 (Fig. 1).

### Data Collection

We collected the following information from the patient’s medical records: basic demographic features, tumor-related characteristics (tumor clinical stage, local invasion [invasion of the chest wall and/or skin], lymphovascular invasion [LVI], pathological type, histological grade, Ki67, estrogen receptor [ER], progesterone receptor [PR], HER2, and hemoglobin level), treatment-related data (NCT regimen and times), and pathological outcome.

The patients in our cohort received an NCT regimen consisting of docetaxel + epirubicin + cyclophosphamide (TEC) or docetaxel + carboplatin + trastuzumab (TCbH) every 3 weeks before surgery.^17^ The following features were considered categorical variables: clinical tumor size, as assessed using ultrasound (categorized as T1 ≤ 2 cm, 2 cm < T2 ≤ 5 cm, T3 > 5 cm); multifocal tumors were categorized as multifocal or unifocal; pathological types were categorized as invasive ductal carcinoma (IDC) and others (invasive lobular carcinoma, other types of invasive carcinoma); and diagnostic biopsy and resected specimens were evaluated by a dedicated breast pathologist.

Immunohistochemical (IHC) staining was used to assess the expression of ER, PR, HER2, and Ki67 in tissues. An ER and PR expression level of < 1% by IHC was considered negative.^18^ The absence of both ER and PR was defined as HR-negative (HR−), and the presence of either was defined as HR-positive (HR+). HER2 positivity was defined as 3(+) according to IHC analysis or amplification confirmed by fluorescence in situ hybridization (FISH); lower scores were defined as HER2-negative (HER2−).^19^ Ki67 expression was divided into two groups: Ki67 > 30% and Ki67 ≤ 30%;^20^ and molecular subtypes were divided into three categories: luminal A subtype (ER-positive [ER+] and/or PR-positive [PR+], any HER2−, Ki67 ≤ 30%); luminal B subtype (ER+ and/or PR+, any HER2 status, Ki67 > 30%), HER2+ subtype (ER-negative [ER−], PR-negative [PR−], HER2+), and triple-negative subtype (ER−, PR−, HER2−).^21^ Considering the limited benefits from NCT for luminal A breast cancer, we did not include it in the neoadjuvant population. Our model is to predict the pathological outcomes of primary breast lesions to NCT based on the Miller/Payne (MP) grading system.^22^ MP1-2 is defined as stable disease/progressive disease (SD/PD), i.e. non-sensitive to chemotherapy, and MP3-5 is defined as partial remission/complete remission (PR/CR), i.e. sensitive to chemotherapy. The objective remission rate (ORR) is generally defined as the sum of the PR plus the CR.

### Statistical Analysis

We used the Chi square test or Fisher’s exact test to evaluate the distribution of basic characteristics of patients among different groups. To develop a well-calibrated nomogram to predict the pathological outcomes, we performed univariate and multivariate logistic regression analyses to screen for predictors.^23^ Using the clinical and pathological data of the training cohort, univariate logistic regression analysis was performed to explore pathological response-related variables. Subsequently, multivariate logistic regression analysis was used to determine the variables that were independent influencing factors of pathological outcomes and to establish the nomogram of the prediction model. The nomogram was validated internally in the training set and externally in the validation set. Internal validation was performed using a calibration method and the area under the receiver operating characteristic (ROC) curve (AUC). External validation was performed by calculating the AUC. The AUC ranged from 0 to 1, with 1 indicating perfect concordance, 0.5 indicating no better than chance, and 0 indicating discordance. A calibration plot with bootstrapping was used to illustrate the association between the actual probability and the predicted probability.^24^ The goodness-of-fit of the model was assessed using the Hosmer and Lemeshow test, with *p* > 0.05 indicating a good fit,^25^ The odds ratios (ORs) and 95% confidence intervals (CIs) were also calculated. The diagnostic odds ratio (DOR) was calculated to further evaluate the performance of the nomogram, and ranges from 0 to infinity (with higher values indicating better performance of a discriminatory test). A value of 1 means that a test does not discriminate between patients with the disorder and those without.^26^

Statistical analyses were performed using IBM SPSS Statistics 24.0 software (IBM Corporation, Armonk, NY, USA) and R version 3.3.3 software (The R Foundation for Statistical Computing, Austria, Vienna). A *p* value < 0.05 was deemed statistically significant.

## Results

### Patient Characteristics

In this research, we retrospectively collected data for 165 patients with operable breast cancer who underwent NCT, including the primary (*n* = 110) and validation (*n* = 55) cohorts, and summarized their demographic and clinicopathological characteristics (Table 1). Among these patients, 128 (77.58%) showed an effective response (PR/CR) after NCT. The ORRs of NCT in the training and validation cohorts were 72.73% and 87.27%, respectively.

**Table 1:** Clinicopathological characteristics of the training and validation cohorts

Variables	Entire cohort	Training cohort	Validation cohort
	[*n* (%)]	[*n* (%)]	[*n* (%)]
No. of patients	165	110	55
Age, years			
≤ 45	63 (38.18)	41 (37.27)	22 (40.00)
> 45	102 (61.82)	69 (62.73)	33 (60.00)
BMI, kg/m^2^			
< 25	121 (73.33)	82 (74.55)	39 (70.91)
≥ 25	44 (26.67)	28 (25.45)	16 (29.09)
Menopausal status			
Postmenopausal	77 (46.67)	51 (46.36)	26 (47.27)
Premenopausal	88 (53.33)	59 (53.64)	29 (52.73)
Multifocality			
Multifocal	37 (22.42)	24 (21.82)	13 (23.64)
Unifocal	128 (77.58)	86 (78.18)	42 (76.36)
Local invasion			
Yes	7 (4.24)	6 (5.45)	1 (1.82)
No	158 (95.76)	104 (94.55)	54 (98.18)
LVI			
Absent	124 (75.15)	83 (75.45)	41 (74.55)
Present	41 (24.85)	27 (24.55)	14 (25.45)
Clinical tumor size			
T1	19 (11.52)	13 (11.82)	6 (10.91)
T2	123 (74.55)	82 (74.55)	41 (74.55)
T3	23 (13.94)	15 (13.64)	8 (14.55)
Lymph node status			
N0	52 (31.52)	37 (33.64)	15 (27.27)
N1-2	113 (68.48)	73 (66.36)	40 (72.73)
Histological grade			
I–II	53 (32.12)	40 (36.36)	13 (23.64)
III	112 (67.88)	70 (63.64)	42 (76.36)
Histological type			
IDC	152 (92.12)	102 (92.73)	50 (90.91)
Others	13 (7.88)	8 (7.27)	5 (9.09)
Estrogen receptor			
Negative	83 (50.30)	57 (51.82)	26 (47.27)
Positive	82 (49.70)	53 (48.18)	29 (52.73)
Progesterone receptor			
Negative	111 (67.27)	74 (67.27)	37 (67.27)
Positive	54 (32.73)	36 (32.73)	18 (32.73)
Hormone receptor			
Negative	82 (49.70)	56 (50.91)	26 (47.27)
Positive	83 (50.30)	54 (49.09)	29 (52.73)
HER2			
Negative	84 (50.91)	55 (50.00)	29 (52.73)
Positive	81 (49.09)	55 (50.00)	26 (47.27)
Ki67			
≤ 30	131 (79.39)	91 (82.73)	40 (72.73)
> 30	34 (20.61)	19 (17.27)	15 (27.27)
Biological subtype			
Luminal B	45 (27.27)	29 (26.36)	16 (29.09)
HER2-enriched	69 (41.82)	47 (42.73)	22 (40.00)
Triple-negative	51 (30.91)	34 (30.91)	17 (30.91)
NCT regimen			
TEC	86 (52.12)	57 (51.82)	29 (52.73)
TCbH	79 (47.88)	53 (48.18)	26 (47.27)
NCT time			
≤ 4	28 (16.97)	23 (20.91)	5 (9.09)
> 4	137 (83.03)	87 (79.09)	50 (90.91)
Hemoglobin, g/L			
< 110	14 (8.48)	9 (8.18)	5 (9.09)
≥ 110	151 (91.52)	101 (91.82)	50 (90.91)
MP stage			
SD/PD	37 (22.42)	30 (27.27)	7 (12.73)
PR/CR	128 (77.58)	80 (72.73)	48 (87.27)

*BMI* body mass index, *HER2* human epidermal growth factor receptor 2, *IDC* intraductal carcinoma, *LVI* lymphovascular invasion, *NCT* neoadjuvant chemotherapy, *MP* Miller/Payne grading system, *SD/PD* stable disease/progressive disease, *PR/CR* partial remission/complete remission, *TEC* docetaxel + epirubicin + cyclophosphamide, *TCbH* docetaxel + carboplatin + trastuzumab

### Predictors for the Effectiveness of Neoadjuvant Chemotherapy (NCT)

The results of univariate and multivariate logistic regression analysis in the training cohort are shown in Table 2. We found that factors related to the efficacy of NCT for patients with breast cancer included age, LVI, ER, HR, biological subtype, and NCT cycles. Considering that the correlation between ER, HR, and biological subtype might make the multifactor model inaccurate, we eliminated HR and biological subtype from the multivariate logistic regression analysis. In addition, considering the importance of HER2 in previous studies, we added HER2 to the multifactor model through stepwise regression analysis. According to the results, younger patients (≤ 45 years of age) are more inclined to show a CR/PR than elderly patients (*p* = 0.011; OR 0.191, 95% CI 0.053–0.680). Patients receiving more than four cycles of NCT have higher efficiency than patients receiving less than four cycles (*p* < 0.001; OR 0.060, 95% CI 0.014–0.250), and patients with LVI were associated with lower efficiency (*p* = 0.045; OR 3.970, 95% CI 2.355–8.382). Patients with ER+ breast cancer were less likely to achieve CR/PR than patients with ER− disease (*p* < 0.001; OR 0.093, 95% CI 0.026–0.334). In contrast, patients with HER2+ disease were more likely to achieve CR/PR than patients with HER2− disease (*p* = 0.029; OR 3.569, 95% CI 1.142–11.151).

**Table 2:** Univariate and multivariate logistic regression analysis for different variables predicting pathological outcomes in the training cohort

Variables	Univariate analysis			Multivariate analysis		
	OR	95% CI	*p* value	OR	95% CI	*p* value
Age, years						
≤ 45	1			1		
> 45	0.412	0.158–1.070	0.044	0.191	0.053–0.680	0.011
Hemoglobin, g/L						
≤ 110	1					
> 110	0.730	0.170–3.124	0.671			
BMI, kg/m^2^						
≥ 25	1					
< 25	1.376	0.540–3.506	0.504			
Multifocality						
Unifocal	1					
Multifocal	0.538	0.206–1.408	0.207			
Menopausal status						
Premenopausal	1					
Postmenopausal	0.565	0.242–1.319	0.187			
Local invasion						
No	1					
Yes	0.867	0.645–5.575	0.246			
LVI						
Present	1			1		
Absent	1.897	1.697–3.963	0.026	3.970	2.355–8.382	0.045
Clinical tumor size						
T1	1					
T2	0.871	0.219–3.472	0.594			
T3	0.450	0.086–2.349	0.250			
Lymph node status						
N0	1					
N1-2	1.202	0.500–2.892	0.681			
Histological grade						
I–II	1					
III	1.501	0.636–3.540	0.353			
Histological type						
Others	1		–			
IDC	0.881	0.168–4.626	0.881			
Progesterone receptor						
Negative	1		–			
Positive	0.433	0.182–1.034	0.059			
Estrogen receptor	–		–			
Negative	1			1		
Positive	0.286	0.116–0.703	0.006	0.093	0.026–0.334	0.000
Hormone receptor						
Negative	1					
Positive	0.301	0.122–0.739	0.009			
HER2						
Negative	1			1		
Positive	1.743	0.743–4.088	0.201	3.569	1.142–11.151	0.029
Ki67	–		–			
≤ 30	1		–			
> 30	0.776	0.265–2.271	0.644			
Biological subtype	–		–			
HER2-enriched	1		–			
Luminal B	0.333	0.121–0.915	0.016			
Triple-negative	1.042	0.352–3.088	0.233			
NCT regimen	–		–			
TEC	1					
TCbH	1.577	0.673–3.696	0.295			
NCT times	–		–			
> 4	1		–	1		
≤ 4	0.187	0.070–0.498	0.001	0.060	0.014–0.250	0.000

*OR* odds ratio, *CI* confidence interval, *BMI* body mass index, *HER2* human epidermal growth factor receptor 2, *LVI* lymphovascular invasion, *NCT* neoadjuvant chemotherapy, *TEC* docetaxel + epirubicin + cyclophosphamide, *TCbH* docetaxel + carboplatin + trastuzumab, *IDC* intraductal carcinoma

### Establishment and Validation of the Nomogram

**Fig. 2:**
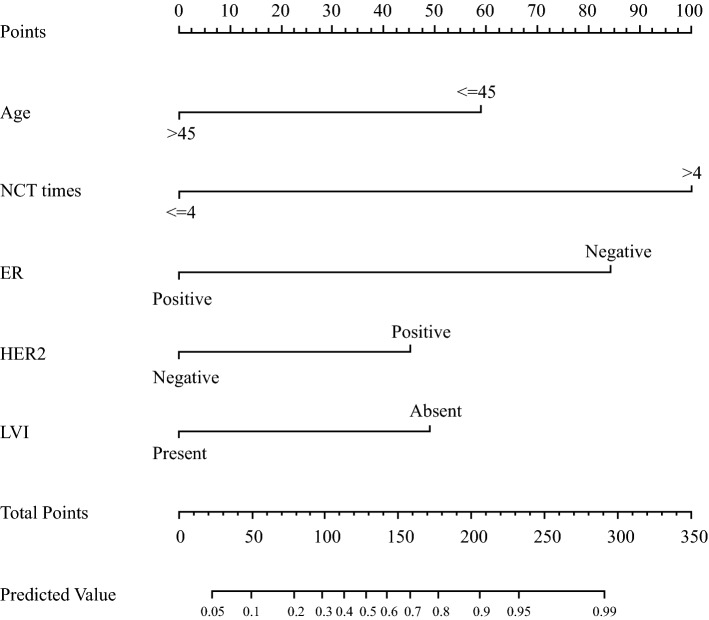
Nomogram to predict the pathological outcomes of NCT. *HER2* human epidermal growth factor receptor 2, *LVI* lymphovascular invasion, *NCT* neoadjuvant chemotherapy, *ER* estrogen receptor

**Fig. 3:**
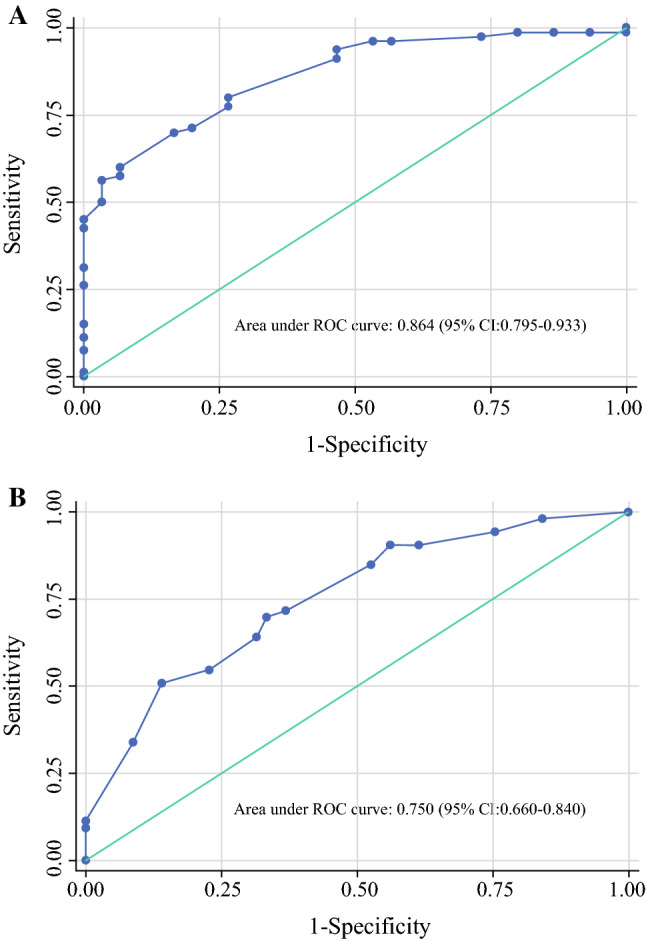
Area under the receiver operating characteristic curves of the nomogram to predict the pathological outcomes of NCT in the (**a**) training and (**b**) validation cohorts. *ROC* receiver operating characteristic, *CI* confidence interval, *NCT* neoadjuvant chemotherapy

**Fig. 4:**
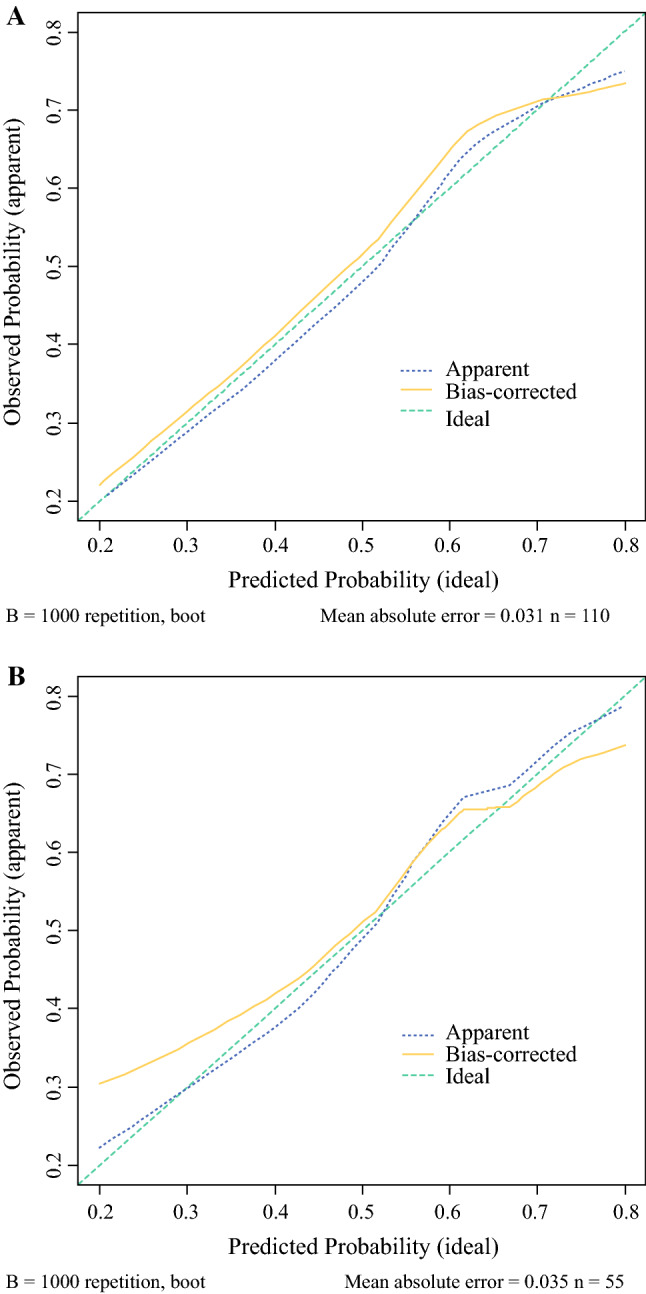
Calibration of the nomogram to predict the pathological outcomes of NCT in the (**a**) training and (**b**) validation cohorts. *NCT* neoadjuvant chemotherapy

Based on the independent predictors identified in the multivariate logistic regression analysis, a nomogram including age, NCT cycles, ER, HER2, and LVI to predict the pathological outcomes after NCT for patients with breast cancer was developed (Fig. 2). The corresponding scores for the following factors (top plotting scale) were summed up to the total points, which corresponded to the predicted value of the ORR (bottom plotting scale): age, in years (≤ 45, 59; > 45, 0), NCT cycles (≤ 4, 0; > 4, 100), ER (negative, 84; positive, 0), HER2 (negative, 0; positive, 45), and LVI (present, 0; absent, 49). Eventually, the predicted value of pathological outcomes was expressed by the following equation (Eq. 1):

1 $$\begin{aligned} & n\left( {p/1 - p} \right) = 3.251 - 1.658 \times a - 2.372 \times b \\ & \quad + 1.272 \times c + 1.379 \times d - 2.813 \times e \\ \end{aligned}$$

where ‘*p*’ represents the predicted value of CR/PR, ‘*a*’ represents age at diagnosis, ‘*b*’ represents ER, ‘*c*’ represents HER2, ‘*d*’ represents LVI; and ‘*e*’ represents NCT cycles. ROC analysis was performed to validate the nomogram internally in the training cohort (Fig. 3a) and externally in the validation cohort (Fig. 3b), with AUC values of 0.864 (95% CI 0.795–0.933) and 0.750 (95% CI 0.660–0.840), respectively, suggesting that it had a good predictive ability. The calibration of the nomogram was performed internally in the training cohort (Fig. 4a) and externally in the validation cohort (Fig. 4b), using a calibration plot with bootstrap sampling (*n* = 1000). There was satisfactory agreement between the predicted probability and the observed probability, according to an administered Hosmer–Lemeshow test (Hosmer–Lemeshow test in the training cohort: Chi square = 3.386, *p* = 0.908; Hosmer–Lemeshow test in the validation cohort: Chi square = 2.784, *p* = 0.972).

### Determining the Cut-Off Value for Predicting Pathological Outcomes After NCT

**Fig. 5:**
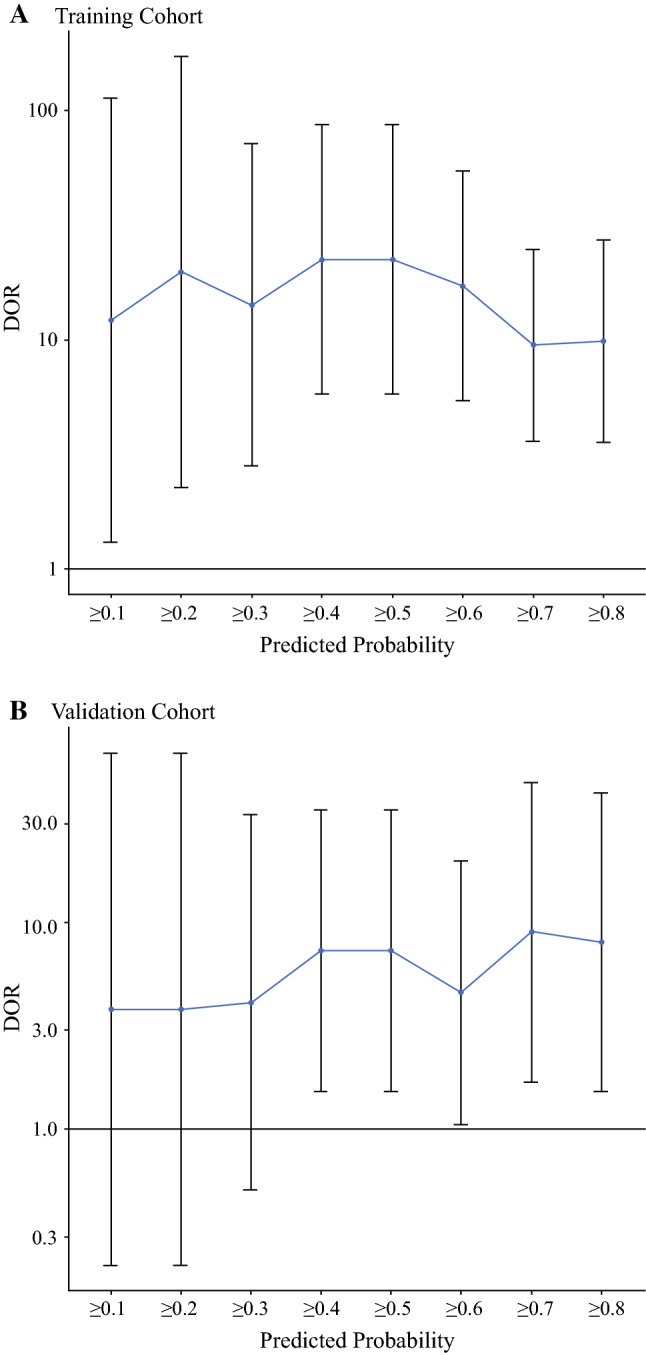
The DOR of the nomogram to predict the pathological outcomes of NCT in the (**a**) training cohort and (**b**) validation cohort at different cut-off values. *DOR* diagnostic odds ratio, *NCT* neoadjuvant chemotherapy

Using different cut-off values of the nomogram, the values of sensitivity, specificity, and predictive values of the predicted probability were calculated (Table 3). A higher cut-off value resulted in an increase in specificity and positive predictive value, and a decrease in sensitivity and negative predictive value. The DORs of the nomogram at different cut-off values are shown in Table 4. The cut-off values for good performance of the nomogram ranged from ≥ 0.1 to ≥ 0.8 in the training cohort (Fig. 5a) and ≥ 0.4 to ≥ 0.8 in the validation cohort (Fig. 5b). According to Youden’s method,^27^ the optimal cut-off value was 0.646 (in the training cohort: sensitivity, 80%; specificity, 73.3%; positive predictive value, 88.9%; negative predictive value, 57.9%; Youden Index, 53.3%; and in the validation cohort: sensitivity, 69.2%; specificity, 81.8%; positive predictive value, 93.1%; negative predictive value, 42.9%; Youden Index, 51%) [Table 5].

**Table 3:** Values for sensitivity, specificity, PPV, NPV, and Youden index of the predicted probability at different cut-off values

Predicted probability	Training cohorts					Validation cohorts				
	Sensitivity (%)	Specificity (%)	PPV (%)	NPV (%)	Index	Sensitivity (%)	Specificity (%)	PPV (%)	NPV (%)	Index
0.126	98.8	13.3	75.2	80.0	12.1	97.4	0.0	77.6	0.0	− 2.6
0.228	98.8	20.0	76.7	85.7	18.8	97.4	9.1	79.2	50.0	6.5
0.315	97.5	26.7	78.0	80.0	24.2	94.9	18.2	80.4	50.0	13.1
0.465	96.3	43.3	81.9	81.3	39.6	89.7	36.4	83.3	50.0	26.1
0.513	96.3	46.7	82.8	82.4	42.9	89.7	45.5	85.4	55.6	35.2
0.608	93.8	53.3	84.3	76.2	47.1	84.6	45.5	84.6	45.5	30.1
0.646	80.0	73.3	88.9	57.9	53.3	69.2	81.8	93.1	42.9	51.0
0.707	77.5	73.3	88.6	55.0	50.8	66.7	81.8	92.9	40.9	48.5
0.896	50.0	96.7	97.6	42.0	46.7	41.0	90.9	94.1	30.3	31.9

*PPV* positive predictive value, *NPV* negative predictive value, *Index* Youden index

**Table 4:** The diagnostic odds ratios of the nomogram at different cut-off values

Predicted probability	DOR (95% CI)	
	Training cohorts	Validation cohorts
≥0.1	12.154 (1.299–113.676)	3.8 (0.218–66.225)
≥0.2	19.75 (2.265–172.246)	3.8 (0.218–66.225)
≥0.3	14.182 (2.806–71.672)	4.111 (0.508–33.271)
≥0.4	22.458 (5.774–87.352)	7.292 (1.51–35.203)
≥0.5	22.458 (5.774–87.352)	7.292 (1.51–35.203)
≥0.6	17.143 (5.401–54.4120	4.583 (1.052–19.964)
≥0.7	9.472 (3.611–24.849)	9 (1.693–47.838)
≥0.8	9.913 (3.585–27.415)	8.036 (1.519–42.519)

*DORs* diagnostic odds ratios

**Table 5:** Values for sensitivity, specificity, PPV and NPV of the predicted probability at the optimal cutoff value

	Optimal cut-off	Sensitivity (%)	Specificity (%)	PPV (%)	NPV (%)
Training set	0.646	80.0	73.3	88.9	57.9
Validation set	0.646	69.2	81.8	93.1	42.9

*PPV* positive predictive value, *NPV* negative predictive value

### Prospective Applications of the Nomogram

To demonstrate the application of the model, we selected as examples two breast cancer patients who received NCT. For patient 1 (38 years of age; 59 points), hollow needle puncture confirmed IDC of the right breast. IHC suggested ER (−) (84 points), PR (−), HER2 (−) [0 points], Ki67 (+80%), triple-negative subtype D2-40 (+), with LVI (0), and six-cycle TEC protocol NCT (100 points), giving a final score of 243 and a predicted value of an ORR of 0.97, which was > 0.646. This patient is very likely to achieve CR/PR after NCT and her postoperative pathological status was CR. For patient 2 (58 years of age; 0 points), hollow needle puncture confirmed IDC of the left breast. IHC suggested ER (+, 80%) [0 points], PR (+, 50%), HER2 (−) [0 points], Ki67 (+30%), luminal B, HER2− subtype, D2-40 (−), no LVI (0 points), and six-cycle TEC protocol NCT (100), giving a final score of 100 and a predicted value of an ORR was 0.32, which was < 0.646. This patient is very likely to have a poor response to NCT, and her postoperative pathological status was SD.

## Discussion

NCT shows diverse efficacy among patients with breast cancer. Therefore, exploring accurate methods to screen patients who can benefit from NCT before treatment has become a hot research topic in recent years. Various investigations have focused on predicting the efficacy of NCT in patients with breast cancer, including the analysis of inflammatory markers,^28^ application of histomorphological factors,^29^ analysis of molecular biomarkers,^30^ and the use of medical imaging indicators.^31^^,^^32^ However, given the heterogeneity of tumors, the accuracy of predicting efficacy using a single factor is limited; therefore, researchers have attempted to predict the efficacy of NCT using a multifactorial model. Takada et al. developed a prediction model for pCR after NCT using ADTree,^8^ in which the fluorouracil, epirubicin, and cyclophosphamide (FEC) regimen was used for the whole study population; however, they failed to take into account the use of targeted drugs in patients with HER2+ breast cancer. Fujii et al. constructed a nomogram to predict pCR in HER2+ breast cancer treated with NCT regimens containing trastuzumab;^12^ however, the C-index of the nomogram was only 0.69 and they did not have an independent cohort to validate the nomogram.

In the present study, we first developed a nomogram to provide early prediction of the response to NCT in breast cancer based on five preoperative covariates: age, NCT cycles, ER, HER2, and LVI. The nomogram was validated internally in the training set and externally in the validation set, with AUC values of 0.864 (95% CI 0.795–0.933) and 0.750 (95% CI 0.660–0.840), respectively. The model exhibited sufficient ability to predict the pathological response of NCT among patients with breast cancer. Finally, according to the Youden index and the DORs, we assigned an optimal cut-off value of 0.646.

As described in the results, univariate analysis showed that age, LVI, ER, HR, molecular subtypes, and NCT cycles were related to the efficacy of NCT; however, before including these factors in the multivariate analysis, the reproducibility among the ER, HR, and molecular subtypes was assessed for their effects on the final results, and only age, LVI, ER, and NCT cycles were included in the multivariate analysis. At the same time, considering the importance of HER2 status, we incorporated it into the model using the stepwise regression method. Ultimately, patients who were younger (≤ 45 years of age), received more than four cycles of NCT, in whom LVI was absent, and who were ER− and HER2+ were more likely to benefit from NCT than other patients.

In previous studies, the relationship between age and the efficacy of NCT for primary breast cancer lesions was rarely mentioned. Some studies have found that young patients achieve pCR more easily after NCT in axillary lymph node lesions,^33^^–^35 which is consistent with our findings. We suspected that younger patients have a higher degree of malignancy and are more likely to tolerate the aggressive regimens of NCT.

Commonly, the number of NCT cycles is related to efficacy; however, there are still some controversies on how many cycles of NCT should be performed. The NeoSphere^36^ and PEONY^37^ studies conducted four cycles of NCT, the CREATE-X trial^38^ conducted four to eight cycles of NCT, and the TRYPHAENA study^39^ conducted six cycles of NCT; therefore, four to eight cycles of NCT are considered feasible in current clinical practice. Although in some large clinical studies it can be observed that different NCT cycles result in different response rates, no studies have directly compared the effect of chemotherapy cycles on the efficacy of NCT. In our study, we found that patients receiving more than four cycles of NCT showed a higher response rate than patients receiving fewer than four cycles. Therefore, we incorporated NCT cycles into our nomogram, hoping that clinicians and patients can intuitively know the preferred number of NCT cycles through our predictive model prior to treatment.

Previous studies have confirmed that patients with HR− and HER2+ disease are more sensitive to chemotherapy, which is in line with the results of our study.^12^^,^^40^^,^^41^ Furthermore, for the high-risk populations (triple-negative, HER2+), NCT may be the first choice; however, because of tumor heterogeneity, not all HER2+ or triple-negative breast cancer patients respond well to chemotherapy. One of the purposes of our model is to screen out patients with poor response to NCT (SD/PD patients) from these two subtypes before treatment so as to avoid them from missing the best time and options for surgery. If a triple-negative or HER2+ patient with cT2N1 stage is predicted to have a response rate < 0.646, indicating that this patient may have PD/SD during NCT, then the surgery may be of priority. In addition, considering that the nomogram was established based on the retrospective population using conventional NCT regimens, intensive treatment may be recommended according to the results from the CREATE-X^42^ and KATHERINE^43^ studies after operation; however, this requires further verification through clinical research.

Moreover, for breast cancer patients of the HR+/HER2− subtype, whether NCT should be performed is still controversial. Although our model can provide a reference for clinicians’ decision making, the benefits from NCT in these patients are still unclear. Previous studies using genomic testing such as OncotypeDX to predict the efficacy of NCT have not yet reached a unified conclusion. A recent study by Pease et al., using the National Cancer Data Base from 2010 to 2015, found pCR to be associated with high recurrence scores (RS) > 30 (OR 4.87); however, only 4.3% of the ER+/HER2− cohort had pCR.^44^ Soran et al. examined 60 pretreatment core needle biopsies in ER+/HER2− patients who received NCT, with no significant correlation between treatment response and RS.^45^ Thus, OncotypeDx does not seem to be particularly useful for predicting the curative effect of NCT. Additionally, it is not clear whether OncotypeDx results on core biopsy correlate well with OncotypeDx results on a resected specimen because the tissue obtained from core needle biopsy may not be fully reflective of the whole tumor heterogeneity. In future research, we hope to improve the concordance between core needle biopsy tissue and traditional surgery tissue by optimizing the procedure through multifocal needle puncture, and then add genomic testing such as OncotypeDX and MammaPrint to our prediction model through prospective research, so as to predict the benefit of NCT in HR+/HER2− breast cancer patients more accurately.

Notably, LVI is an independent prognostic parameter for poor outcome of invasive breast cancer and is the main prerequisite for metastasis.^46^ A previous study showed that the presence of LVI was significantly associated with chemoresistant breast cancer.^47^ Our previous research showed that LVI is related to low efficiency in patients receiving NCT, further suggesting that LVI is an important molecular target in breast cancer. To the best of our knowledge, this is the first study to incorporate LVI into a model to predict the efficacy of NCT. As we know, Ki67 is a proliferation marker that provides a fast method to assess the proportion of proliferating cells in a tumor.^48^ Previous studies have shown that chemotherapy is more effective in patients with higher Ki67 levels;^49^ however, there was no significant correlation between Ki67 and chemotherapy efficacy in the present study, but this does not mean that Ki67 status is not related to NCT efficacy. One possible explanation is that we did not incorporate patients with the luminal A subtype (ER+ and/or PR+, any HER2−, Ki67 ≤ 30%) in the NCT study population.

Our nomogram has several strengths. First, as far as we know, this is the first study to establish a nomogram to predict the efficacy of neoadjuvant treatment for breast cancer based on clinical pathological data in the past 3 years. Furthermore, the data information upon enrollment was complete. Second, all patients in the study received standard NCT regimens, which complied with the latest version of the National Comprehensive Cancer Network guidelines; therefore, the applicability and representativeness of the nomogram will be stronger. Finally, the clinicopathological variables used in our nomogram are easily assessable using hollow needle biopsy, and the cut-off value that predicts the effectiveness of NCT can be determined before the start of NCT. This nomogram could be used to help surgeons and patients make treatment decisions.

Despite our model providing a promising predictive value in patients with breast cancer receiving NCT, several limitations should be noted. First, although we validated our nomogram using an independent dataset, the validation cohort was extracted from the same institution that produced the training cohort. We will continue to seek external datasets for a future validation study. Second, pertuzumab was approved to enter mainland China in the second half of 2019, and entered the National Medical Insurance system in January 2020; therefore, there was not enough dual-target therapy data to be included in our study. In the future, we hope to add more dual-target therapy cases to our study. Finally, the sample size was relatively small, and the predictive ability of the model needs to be further verified in large-sample studies.

## Conclusion

Our constructed nomogram confirmed that patients who were younger (≤ 45 years of age), were receiving more than four cycles of NCT, in whom LVI was absent, and who were ER− and HER2+ were more likely to respond to NCT than other patients. Using the nomogram, for patients who are operable and whose predicted probability of pathological effectiveness is < 0.646, we believe that the benefits of NCT are limited and these patients can be treated directly using surgery.

## References

[1] Siegel RL, Miller KD (2020). Cancer statistics, 2020. CA: A Cancer Journal for Clinicians.

[2] Golshan M, Cirrincione CT, Sikov WM (2015). Impact of neoadjuvant chemotherapy in stage II–III triple negative breast cancer on eligibility for breast-conserving surgery and breast conservation rates: surgical results from CALGB 40603 (Alliance). Ann Surg..

[3] Generali D, Ardine M, Strina C (2015). Neoadjuvant treatment approach: the Rosetta stone for breast cancer?. J Natl Cancer Inst Monogr..

[4] Cortazar P, Zhang L, Untch M (2014). Pathological complete response and long-term clinical benefit in breast cancer: the CTNeoBC pooled analysis. Lancet..

[5] Tanioka M, Hirokaga K, Kitao A (2012). The clinical significance of pathologic complete response using different definitions after neoadjuvant chemotherapy in HER2 positive breast cancer patients according to hormonal receptor status. Cancer Research..

[6] Hurvitz SA, Martin M, Symmans WF (2018). Neoadjuvant trastuzumab, pertuzumab, and chemotherapy versus trastuzumab emtansine plus pertuzumab in patients with HER2-positive breast cancer (KRISTINE): a randomised, open-label, multicentre, phase 3 trial. The Lancet. Oncology..

[7] Neoadjuvant pembrolizumab takes on TNBC. *Cancer Discov.* 2019;9(10):Of4. 10.1158/2159-8290.CD-NB2019-097 31420357

[8] Takada M, Sugimoto M, Ohno S (2012). Predictions of the pathological response to neoadjuvant chemotherapy in patients with primary breast cancer using a data mining technique. Breast Cancer Res Treat..

[9] Colleoni M, Viale G, Zahrieh D (2008). Expression of ER, PgR, HER1, HER2, and response: a study of preoperative chemotherapy. Ann Oncol..

[10] Goldhirsch A, Wood WC, Coates AS (2011). Strategies for subtypes—dealing with the diversity of breast cancer: highlights of the St Gallen International Expert Consensus on the Primary Therapy of Early Breast Cancer 2011. Ann Oncol..

[11] Zhang F, Huang M, Zhou H (2019). A Nomogram to Predict the Pathologic Complete Response of Neoadjuvant Chemotherapy in Triple-Negative Breast Cancer Based on Simple Laboratory Indicators. Ann Surg Oncol..

[12] Fujii T, Kogawa T, Wu J (2017). Nomogram to predict pathologic complete response in HER2-positive breast cancer treated with neoadjuvant systemic therapy. Br J Cancer..

[13] Zhang F, Zheng W, Ying L (2016). A nomogram to predict brain metastases of resected non-small cell lung cancer patients. Ann Surg Oncol..

[14] Colleoni M, Bagnardi V, Rotmensz N (2010). A nomogram based on the expression of Ki-67, steroid hormone receptors status and number of chemotherapy courses to predict pathological complete remission after preoperative chemotherapy for breast cancer. Eur J Cancer..

[15] Lu C-H, Liu C-T, Chang P-H (2017). Develop and validation a nomogram to predict the recurrent probability in patients with major salivary gland cancer. J Cancer..

[16] Liang W, Zhang L, Jiang G (2015). Development and validation of a nomogram for predicting survival in patients with resected non–small-cell lung cancer. J Clin Oncol..

[17] National Comprehensive Cancer Network. NCCN clinical practice guidelines: breast cancer, version 4. 2017. National Comprehensive Cancer Network; 2018. 10.6004/jnccn.2018.0012 29523670

[18] Hammond MEH, Hayes DF, Dowsett M (2010). American Society of Clinical Oncology/College of American Pathologists guideline recommendations for immunohistochemical testing of estrogen and progesterone receptors in breast cancer (unabridged version). Arch Pathol Lab Med..

[19] Wolff AC, Hammond MEH, Schwartz JN (2007). American Society of Clinical Oncology/College of American Pathologists guideline recommendations for human epidermal growth factor receptor 2 testing in breast cancer. Arch Pathol Lab Med..

[20] Dowsett M, Nielsen TO, A’Hern R (2011). Assessment of Ki67 in breast cancer: recommendations from the International Ki67 in Breast Cancer working group. J Natl Cancer Inst..

[21] Goldhirsch A, Winer EP, Coates A (2013). Personalizing the treatment of women with early breast cancer: highlights of the St Gallen International Expert Consensus on the Primary Therapy of Early Breast Cancer 2013. Ann Oncol..

[22] Ogston KN, Miller ID, Payne S (2003). A new histological grading system to assess response of breast cancers to primary chemotherapy: prognostic significance and survival. Breast..

[23] Rouzier R, Pusztai L, Delaloge S (2005). Nomograms to predict pathologic complete response and metastasis-free survival after preoperative chemotherapy for breast cancer. J Clin Oncol..

[24] Harrell FE, Lee KL, Mark DB (1996). Multivariable prognostic models: issues in developing models, evaluating assumptions and adequacy, and measuring and reducing errors. Stat Med..

[25] Hosmer DW, Lemeshow S, Sturdivant RX (2013). Applied logistic regression.

[26] Glas AS, Lijmer JG, Prins MH, Bonsel GJ, Bossuyt PMM (2003). The diagnostic odds ratio: a single indicator of test performance. J Clin Epidemiol..

[27] Youden WJ (1950). Index for rating diagnostic tests. Cancer..

[28] Zhang F, Huang M, Zhou H, Chen K, Jin J, Wu Y (2019). A Nomogram to Predict the Pathologic Complete Response of Neoadjuvant Chemotherapy in Triple-Negative Breast Cancer Based on Simple Laboratory Indicators. Annals of Surgical Oncology..

[29] Jung YY, Hyun CL, Jin MS (2016). Histomorphological Factors Predicting the Response to Neoadjuvant Chemotherapy in Triple-Negative Breast Cancer. J Breast Cancer..

[30] Li Z, Zhang Y, Zhang Z, Zhao Z, Lv Q (2019). A four-gene signature predicts the efficacy of paclitaxel-based neoadjuvant therapy in human epidermal growth factor receptor 2-negative breast cancer. J Cell Biochem.

[31] Liu Z, Li Z, Qu J, Zhang R, Zhou X, Li L (2019). Radiomics of multi-parametric MRI for pretreatment prediction of pathological complete response to neoadjuvant chemotherapy in breast cancer: a multicenter study. Clinical Cancer Research..

[32] Baumgartner A, Tausch C, Hosch S (2018). Ultrasound-based prediction of pathologic response to neoadjuvant chemotherapy in breast cancer patients. Breast..

[33] Alvarado R, Yi M, Le-Petross H (2012). The role for sentinel lymph node dissection after neoadjuvant chemotherapy in patients who present with node-positive breast cancer. Ann Surg. Oncol..

[34] Koolen BB, Valdés Olmos RA, Wesseling J, Vogel WV, Vincent AD, Gilhuijs KGA (2013). Early Assessment of Axillary Response with ^18^F-FDG PET/CT during Neoadjuvant Chemotherapy in Stage II–III Breast Cancer: Implications for Surgical Management of the Axilla. Ann Surg Oncol..

[35] Liu C, Jiang Y, Gu X, Xu Z (2017). Predicting level 2 axillary lymph node metastasis in a Chinese breast cancer population post-neoadjuvant chemotherapy: Development and assessment of a new predictive nomogram. Oncotarget.

[36] Gianni L, Pienkowski T, Im Y-H (2012). Efficacy and safety of neoadjuvant pertuzumab and trastuzumab in women with locally advanced, inflammatory, or early HER2-positive breast cancer (NeoSphere): a randomised multicentre, open-label, phase 2 trial. The Lancet Oncology..

[37] Shao Z, Pang D, Yang H (2020). Efficacy, Safety, and Tolerability of Pertuzumab, Trastuzumab, and Docetaxel for Patients With Early or Locally Advanced ERBB2-Positive Breast Cancer in Asia: The PEONY Phase 3 Randomized Clinical Trial. JAMA oncology..

[38] Masuda N, Lee SJ, Ohtani S (2017). Adjuvant Capecitabine for Breast Cancer after Preoperative Chemotherapy. The New England journal of medicine..

[39] Schneeweiss A, Chia S, Hickish T (2013). Pertuzumab plus trastuzumab in combination with standard neoadjuvant anthracycline-containing and anthracycline-free chemotherapy regimens in patients with HER2-positive early breast cancer: a randomized phase II cardiac safety study (TRYPHAENA). Annals of oncology..

[40] Caudle AS, Gonzalez-Angulo AM, Hunt KK (2010). Predictors of Tumor Progression During Neoadjuvant Chemotherapy in Breast Cancer. J Clin Oncol..

[41] Jin X, Jiang Y-Z, Chen S, Shao Z-M, Di G-H (2016). A nomogram for predicting the pathological response of axillary lymph node metastasis in breast cancer patients. Sci Rep..

[42] Masuda N, Lee SJ, Ohtani S (2017). Adjuvant Capecitabine for Breast Cancer after Preoperative Chemotherapy. N Engl J Med..

[43] von Minckwitz G, Huang CS, Mano MS (2019). Trastuzumab Emtansine for Residual Invasive HER2-Positive Breast Cancer. The New England journal of medicine..

[44] Pease AM, Riba LA, Gruner RA, Tung NM (2019). James TA Oncotype DX^®^ Recurrence Score as a Predictor of Response to Neoadjuvant Chemotherapy. Ann Surg Oncol..

[45] Soran A, Bhargava R, Johnson R (2016). The impact of Oncotype DX^®^ recurrence score of paraffin-embedded core biopsy tissues in predicting response to neoadjuvant chemotherapy in women with breast cancer. Breast disease..

[46] Lee AHS, Pinder SE, Macmillan RD (2006). Prognostic value of lymphovascular invasion in women with lymph node negative invasive breast carcinoma. Eur J Cancer..

[47] Uematsu T, Kasami M, Watanabe J (2011). Is lymphovascular invasion degree one of the important factors to predict neoadjuvant chemotherapy efficacy in breast cancer?. Breast Cancer..

[48] Gerdes J, Schwab U, Lemke H, Stein H (1983). Production of a mouse monoclonal antibody reactive with a human nuclear antigen associated with cell proliferation. Int J Cancer..

[49] Denkert C, Loibl S, Müller BM (2013). Ki67 levels as predictive and prognostic parameters in pretherapeutic breast cancer core biopsies: a translational investigation in the neoadjuvant GeparTrio trial. Ann Oncol..

